# Functional and structural studies of the vaccinia virus virulence factor N1 reveal a Bcl-2-like anti-apoptotic protein

**DOI:** 10.1099/vir.0.82772-0

**Published:** 2007-06

**Authors:** Samantha Cooray, Mohammad W. Bahar, Nicola G. A. Abrescia, Colin E. McVey, Nathan W. Bartlett, Ron A.-J. Chen, David I. Stuart, Jonathan M. Grimes, Geoffrey L. Smith

**Affiliations:** 1 Department of Virology, Faculty of Medicine, Imperial College London, St Mary's Campus, Norfolk Place, London W2 1PG, UK; 2 The Oxford Protein Production Facility and The Division of Structural Biology, Wellcome Trust Centre for Human Genetics, University of Oxford, Roosevelt Drive, Oxford OX3 7BN, UK

## Abstract

Vaccinia virus (VACV) encodes many immunomodulatory proteins, including inhibitors of apoptosis and modulators of innate immune signalling. VACV protein N1 is an intracellular homodimer that contributes to virus virulence and was reported to inhibit nuclear factor (NF)-*κ*B signalling. However, analysis of NF-*κ*B signalling in cells infected with recombinant viruses with or without the *N1L* gene showed no difference in NF-*κ*B-dependent gene expression. Given that N1 promotes virus virulence, other possible functions of N1 were investigated and this revealed that N1 is an inhibitor of apoptosis in cells transfected with the *N1L* gene and in the context of VACV infection. In support of this finding virally expressed N1 co-precipitated with endogenous pro-apoptotic Bcl-2 proteins Bid, Bad and Bax as well as with Bad and Bax expressed by transfection. In addition, the crystal structure of N1 was solved to 2.9 Å resolution (0.29 nm). Remarkably, although N1 shows no sequence similarity to cellular proteins, its three-dimensional structure closely resembles Bcl-x_L_ and other members of the Bcl-2 protein family. The structure also reveals that N1 has a constitutively open surface groove similar to the grooves of other anti-apoptotic Bcl-2 proteins, which bind the BH3 motifs of pro-apoptotic Bcl-2 family members. Molecular modelling of BH3 peptides into the N1 surface groove, together with analysis of their physico-chemical properties, suggests a mechanism for the specificity of peptide recognition. This study illustrates the importance of the evolutionary conservation of structure, rather than sequence, in protein function and reveals a novel anti-apoptotic protein from orthopoxviruses.

## INTRODUCTION

Apoptosis represents an important host-innate immune response to infection and aids elimination of virus-infected cells (Roulston *et al.*, 1999). The B-cell lymphoma protein-2 (Bcl-2) family regulates the release of pro-apoptotic molecules from mitochondria and includes both pro- and anti-apoptotic members. These contain one or more distinct Bcl-2 homology motifs (BH1–4) and, in some cases, an additional C-terminal hydrophobic helix for targeting the protein to intracellular membranes (Cory *et al.*, 2003). Anti-apoptotic members such as Bcl-x_L_, Bcl-2, Bcl-w and Mcl-1 contain several BH motifs, whereas pro-apoptotic members, such as Bak, Bax and Bok, may contain multiple BH motifs, or may be BH3 ‘only’ such as Bad, Bid, Bim, Hrk, Noxa and Puma (Cory *et al.*, 2003). The crystal structure of Bcl-x_L_ and the nuclear magnetic resonance structure of other anti-apoptotic Bcl-2 proteins show a surface groove that binds BH3 motifs of pro-apoptotic family members (Petros *et al.*, 2004).

Apoptotic signalling pathways are evolutionarily conserved in eukaryotes and Bcl-2 counterparts are present in *Drosophila melanogaster*, *Caenorhabditis elegans* and mammals. During their evolution viruses have developed mechanisms to block apoptosis, permitting completion of their replication cycle. Several DNA viruses encode anti-apoptotic Bcl-2-like proteins that were identified through the presence of BH motifs in the primary amino acid sequence (Polster *et al.*, 2004). Bcl-2-like proteins are encoded by gammaherpesviruses Epstein–Barr virus (Hickish *et al.*, 1994), Kaposi's sarcoma-associated herpesvirus (Sarid *et al.*, 1997) and murine gammaherpesvirus 68 (Virgin *et al.*, 1997), by the alphaherpesvirus Marek's disease virus (Afonso et al., 2001) and also by African swine fever virus (Neilan *et al.*, 1993). The genomes of orthopoxviruses such as VACV and variola virus, the causative agent of smallpox do not encode proteins with identifiable BH motifs. However, Bcl-2 counterparts would perhaps be expected given that poxviruses encode numerous immunomodulatory proteins, including serpins, decoy receptors for interleukin (IL)-1*β*, interferon (IFN)-*γ*, IFN-*β* and tumour necrosis factor (TNF)-*α* (Seet *et al.*, 2003), and employ several anti-apoptotic strategies (for review see Taylor & Barry, 2006). In addition, avipoxviruses such as fowlpox and canarypox contain identifiable Mcl-1 counterparts (Afonso *et al.*, 2000; Tulman *et al.*, 2004), and the VACV F1 protein displays the hallmarks of a functional orthologue as it localizes to mitochondria, inhibits apoptosis and binds pro-apoptotic Bak (Wasilenko *et al.*, 2005; Fischer *et al.*, 2006; Postigo *et al.*, 2006).

The VACV protein N1 was first described as a secreted protein (Kotwal *et al.*, 1989) but was shown subsequently to be a 14 kDa intracellular homodimer that is expressed early in the virus life cycle and contributes to virus virulence in mouse intradermal and intranasal models of infection (Bartlett *et al.*, 2002). Overexpression of N1 was reported to inhibit NF-*κ*B activation downstream of TNF, IL-1 and Toll-like receptors (TLRs) *in vitro*, and this was suggested to be through direct inhibition of the I*κ*B kinase complex (DiPerna *et al.*, 2004). Here, we demonstrate that N1 is an inhibitor of apoptosis. Cells expressing N1 following transfection or virus infection were resistant to staurosporine (ST)-induced apoptosis. Consistent with this, N1 in VACV-infected cells co-precipitated the pro-apoptotic Bcl-2 family proteins Bid, Bad and Bax. Lastly, the crystal structure of N1 was determined and showed compelling structural similarity to Bcl-2 family members, although it lacks amino acid sequence similarity to these proteins. The structure also revealed that N1 contains a surface groove that resembles the BH3-binding grooves of other Bcl-2 proteins. Molecular modelling of BH3 peptides of pro-apoptotic Bcl-2 proteins into the N1 groove suggests a mechanism for binding specificity. These results reveal a Bcl-2-like protein in VACV with anti-apoptotic activity and illustrate the importance of structure in the determination of protein function. During the preparation of this article, the crystal structure of N1 was reported by another group who demonstrated that N1 bound BH3 peptides from pro-apoptotic Bcl-2 family proteins Bid, Bim and Bak *in vitro* (Aoyagi *et al.*, 2007).

## METHODS

### Cells and viruses.

BS-C-1 and HeLa cells were grown at 37 °C in a 5 % CO_2_ atmosphere in Dulbecco's modified Eagle's medium or minimum essential medium (MEM), respectively, supplemented with 10 % heat-treated (56 °C, 1 h) fetal bovine serum (FBS; Invitrogen), 4 mM l-glutamine (Invitrogen), 100 U penicillin (Invitrogen) ml^−1^ and 100 μg streptomycin (Invitrogen) ml^−1^. HeLa cells were also supplemented with MEM non-essential amino acid solution (Sigma-Aldrich). VACV recombinants vN1, vΔN1 and vN1-rev derived from VACV strain Western Reserve (WR) were described previously (Bartlett *et al.*, 2002). Virus infectivity was titrated by plaque assay on BS-C-1 cells.

### Assay for NF-*κ*B activation.

HeLa cells were transfected with a luciferase reporter plasmid, pNF B-Luc, containing NF-*κ*B-binding sites (Stratagene), reseeded and infected with VACV, vΔN1 or vN1-rev at 2 p.f.u. per cell for 2 h. Cells were then treated with 100 ng human IL-1*β* (Peprotech) ml^−1^ for 2 h and lysates were assayed by the luciferase assay system as described by the manufacturer (Promega). Luciferase activity was normalized to the total protein content from the corresponding extract as a transfection efficiency control. Data are expressed as the mean fold induction as a ratio to the mean of the normalized luciferase activity in the mock infection. Two duplicate experiments were carried out with samples in triplicate (Student's *t*-test; *P<0.05; **P<0.005).

### Immunoprecipitation.

HeLa cells were transfected with plasmids expressing HA-tagged Bax, Bad or eiF4E control, and 24 h post-transfection were infected with vN1 or vΔN1 at 10 p.f.u. per cell for 6 h. Alternatively, for immunoprecipitation of endogenous Bcl-2 proteins, HeLa cells were infected for 6 h as above. Monolayers were washed twice in PBS. For analysis of N1 interaction with Bad and Bax expressed by transfection, cells were lysed in CHAPS buffer [100 mM NaCl, 10 mM Tris, pH 7.5, 10 % (w/v) glycerol, 1 % CHAPS, 1 mM MgCl_2_, 1 mM EDTA and 1× protease inhibitor tablet (Roche)]. For analysis of N1 interactions with endogenous levels of Bcl-2 proteins, cells were lysed in HEPES buffer [10 mM HEPES pH 7.4, 150 mM NaCl, 1 mM EDTA, 1 % NP-40, 1× complete protease inhibitor tablet (Roche)]. Lysates were centrifuged at 20 000 ***g*** for 15 min at 4 °C and cytosolic extracts were removed, pre-cleared with protein A- or G-Sepharose beads for 1 h at 4 °C and incubated overnight at 4 °C with mouse monoclonal antibody HA.11 (Cambridge Biosciences) or rabbit polyclonal antibodies to Bax, Bad (Cell Signaling Technology), N1 or Bid (R&D Systems). Immune complexes were bound to protein A– or G–Sepharose beads for 1 h at 4 °C, washed four times in lysis buffer, eluted in 2× loading buffer and boiled. Proteins were separated on NuPAGE Bis-Tris (12 % gel; Invitrogen), transferred to PVDF membranes and blotted with anti-HA.11, anti-N1, anti-*α*-tubulin (Upstate) or anti-Bcl-2 family antibodies. Bound antibody was detected using horseradish peroxidase (HRP)-conjugated anti-rabbit antibodies and visualized using ECL Plus detection (Amersham Biosciences).

### Apoptosis assays.

HeLa cells were mock-infected or infected with wild-type VACV (vN1), vΔN1 or vN1-rev (Bartlett *et al.*, 2002) viruses at 2 p.f.u. per cell for 2 h, or transfected with pCI-based expression vectors for N1, C40S mutant and Bcl-x_L_ together with a CD20 surface marker using Fugene 6 (Roche). Infected cells were stimulated with 1 μM ST for 2 h or left untreated as indicated. Transfected cells were selected using anti-CD20-coated magnetic beads on a magnetic-activated cell sorter (MACS) column 24 h post-transfection. Cells were replated in fresh medium, incubated for a further 24 h at 37 °C and then stimulated with 1 μM ST for 4 h. Cell lysates were tested for caspase 3/7 activity and caspase 3/PARP cleavage using antibodies that detected the cleaved 17 (p17) and 89 kDa (p89) fragments, respectively, as described previously (Cooray *et al.*, 2003). Equivalent loading was verified by blotting for *α*-tubulin (Upstate). Measurement of the change in mitochondrial potential (Δ*ψ*_m_) was carried out using the potentiometric dye JC-1 as described previously (Cossarizza *et al.*, 1993). Briefly, cells were treated with ST, collected, washed in PBS and stained with 2 μM JC-1 dye (Invitrogen) at 37 °C for 30 min. Cells were washed in PBS and stained with anti-CD20 APC antibody (BD Pharmingen) for 20 min on ice. Cells were then washed in PBS, resuspended in FACS buffer [PBS with 2 % (v/v) FBS] and analysed by flow cytometry (FACScan; Becton Dickinson).

### Subcellular fractionation.

Subcellular fractionation was carried out as described previously (Seth *et al.*, 2005). Briefly, HeLa cells were mock-infected or infected with vN1 or vΔN1 at 10 p.f.u. per cell for 6 h. Cells were washed with cold PBS and homogenized by douncing 20 times in 10 mM Tris/HCl pH 7.5, 2 mM MgCl_2_, 10 mM KCl, 250 mM sucrose, 0.5 mM DTT and protease inhibitor cocktail. Nuclei were cleared from homogenates by centrifugation at 500 ***g*** for 10 min at 4 °C. Supernatants were then centrifuged at 5000 ***g*** for 10 min at 4 °C to collect crude mitochondria (M). Supernatants were centrifuged at 15 000 ***g*** to separate cellular organelles/membranes (O) and cytosol (C). Mitochondrial and organelle pellets were resuspended and rehomogenized in lysis buffer. Proteins were separated on NuPAGE Bis-Tris (12 % gel; Invitrogen), transferred to PVDF membranes and blotted with antibodies against N1, cytochrome *c* (BD Biosciences) or Bcl-x_L_ (Cell Signaling Technology). Specific antibody binding was detected using an HRP-conjugated anti-rabbit antibody and visualized using ECL Plus detection.

### Protein expression and crystallization.

The VACV *N1L* gene was cloned into pET24a and expressed as a C-terminally His-tagged protein as described previously (Bartlett *et al.*, 2002). To prevent protein aggregation and heterogeneity, a C40S mutation was introduced using the QuikChange site-directed mutagenesis kit (Stratagene), with primers N1LmutF 5′-GGTAGATGACGGCGATGTAAGCACATTGATTAAGAACTGAGA-3′ and N1LmutR 5′-TCTCATGTTCTTAATCAATGTGCTTACATCGCCGTCATCTACC-3′. Native and selenomethionine (Se-Met) proteins were expressed in *Escherichia* *coli* B834(DE3). For Se-Met incorporation, a single colony was grown overnight in 100 ml base medium (Molecular Dimensions) containing 40 mg l-methionine l^−1^. Bacteria were collected by centrifugation, washed and used to inoculate a 1 l culture containing 40 mg l-selenomethionine l^−1^. Cultures were grown to an absorbance (*A*_600_) of 0.6 and protein expression was induced by the addition of 1 mM IPTG for 16 h at 20 °C.

The mutant N1 protein was purified by nickel-ion-affinity chromatography and gel filtration on a Superdex75 column (Amersham Biosciences). It was then concentrated to ∼14 mg ml^−1^ in 50 mM Tris/HCl pH 8.5, 150 mM NaCl. Crystallization experiments were performed using a Cartesian robot (Brown *et al.*, 2003; Walter *et al.*, 2003). Crystals of N1 mutant were grown at 21 °C by vapour diffusion in 100 nl+100 nl sitting drops equilibrated against 0.2 M Na/K phosphate, 0.2 M NaCl and 10 % PEG 8000, pH 6.2. Crystals belonged to the space group *P*2_1_ (a=71.7 Å, b=109.0 Å, c=70.1 Å, *β*=110.6 °) with six copies of N1 in the asymmetric unit (Table 1). Data were collected at 100 K, using 20 % glycerol as the cryo-protectant.

### Structure determination.

The structure of the N1 C40S mutant protein was solved from a single Se-Met crystal by multiple wavelength anomalous dispersion (MAD). MAD data were collected at BM14 and higher resolution native data on ID23-EH1. Data were processed and reduced using HKL2000 (Table 1) (Otwinowski & Minor, 1997). The positions of the 42 selenium atoms in the asymmetric unit were located and phases were calculated using SOLVE (Terwilliger, 2000). Initial phases were improved by solvent flattening and non-crystallographic symmetry (NCS) averaging using RESOLVE (Terwilliger, 2000).The resulting electron density map allowed a model to be built using COOT (Emsley & Cowtan, 2004). The model was refined, imposing strict sixfold NCS constraints, using CNS against the 2.9 Å resolution data (Brünger *et al.*, 1998). Definition of side chains was improved by sharpening X-ray data with a B-factor of −20 Å^2^. Statistics for the final model are given in Table 1. The model has good stereochemistry and >98 % of residues are within the most favourable regions of the Ramachandran plot (Davis *et al.*, 2004). Molecular representations were created using PyMOL (www.pymol.org).

## RESULTS AND DISCUSSION

**Fig. 1. f1:**
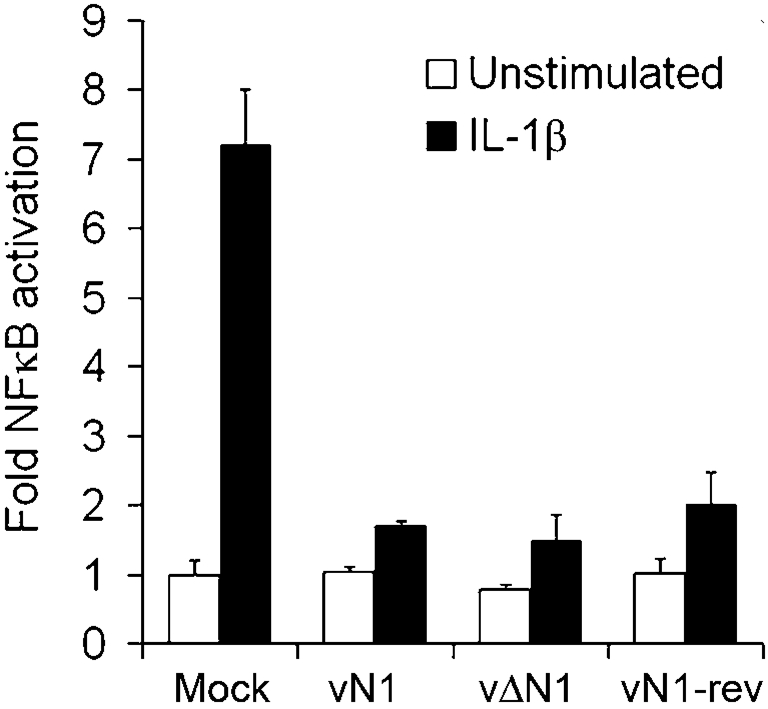
N1 does not inhibit IL-1-induced NF-*κ*B-dependent gene expression in VACV-infected cells. HeLa cells were transfected with the NF-*κ*B luciferase reporter, reseeded and mock-infected or infected with VACV, vΔN1 or vN1-rev at 2 p.f.u. per cell for 2 h. Cells were then treated with 100 ng IL-1*β* ml^−1^ for 2 h and lysates were assayed for luciferase activity. Data are expressed as the mean fold induction compared to the mean of the normalized luciferase activity in the mock infection. Data are means±sd.

Previously, when the N1 protein was overexpressed in transfected cells it was found to inhibit activation of NF-*κ*B following stimulation by IL-1 or Toll-like receptor agonists (DiPerna *et al.*, 2004). Therefore, we extended these studies by analysing if N1 could influence NF-*κ*B-dependent gene expression in VACV-infected cells, where N1 would be expressed at natural levels. HeLa cells were transfected with an NF-*κ*B-responsive reporter gene and subsequently were either mock-infected or infected with a wild-type VACV (vN1), a recombinant VACV engineered to lack the *N1L* gene (vΔN1) or a revertant VACV (vN1-rev) in which the *N1L* gene was reinserted into the deletion mutant at its natural locus (Bartlett *et al.*, 2002). Analysis of NF-*κ*B gene expression showed that in mock-infected cells the addition of IL-1*β* increased gene expression six- to sevenfold, but infection by all three viruses reduced this greatly and to a similar extent (Fig. 1). Therefore, the loss of N1 was not responsible for this virus-induced inhibition of NF-*κ*B-responsive gene expression. This might have been because there are other NF-*κ*B signalling inhibitors encoded by VACV (Haga & Bowie, 2005) that mask a subtle influence of N1 or because N1 exerts its profound effect on virulence by other mechanisms. To address the latter possibility, we investigated if N1 inhibited apoptosis.

### N1 inhibits apoptosis

**Fig. 2. f2:**
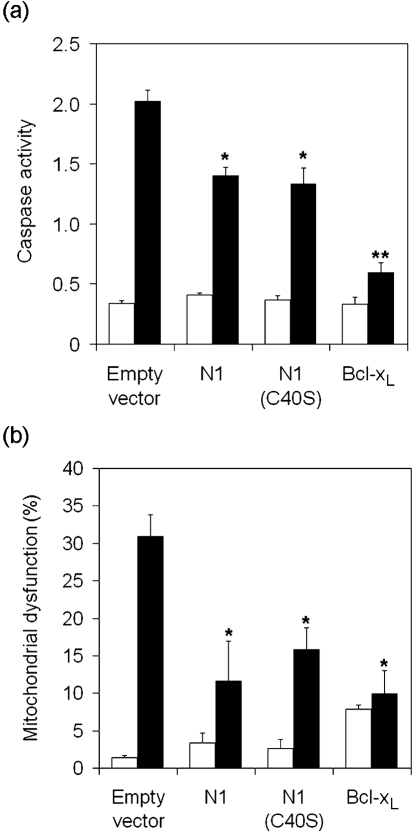
N1 inhibits ST-induced apoptosis in transfected HeLa cells. HeLa cells were transfected with vectors expressing N1, C40S, Bcl-x_L_ or empty control vector (pCI) together with a cell surface CD20 expression vector. After 24 h, transfected cells were selected on MACS columns using anti-CD20-coated magnetic beads as described in Methods and replated into culture medium. After a further 24 h, cells were treated with 1 μM ST (black bars) for 4 h or left untreated (white bars) and assayed for caspase activity (a) and mitochondrial dysfunction (b) as described in Methods. Bcl-x_L_ and Bcl-2 (not shown) were used as positive controls. Asterisks indicate significant difference compared with pCI+ST samples. Data are means±sd (*P<0.05, **P<0.005; Student's *t*-test, *n*=3).

**Fig. 3. f3:**
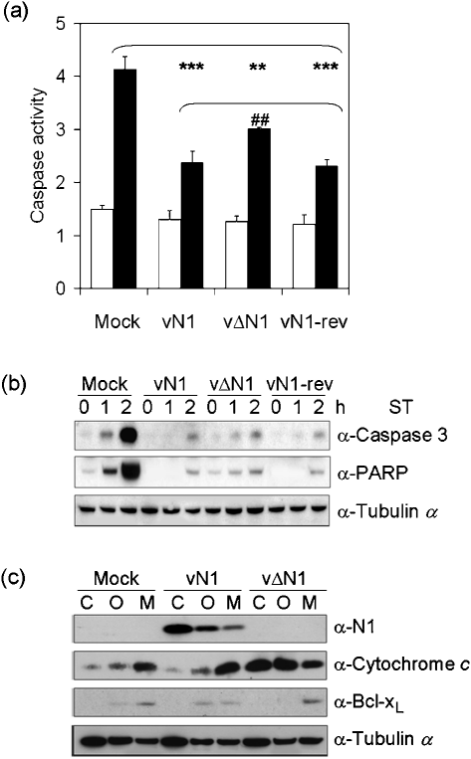
N1 inhibits ST-induced apoptosis in VACV-infected cells. HeLa cells were mock-infected or infected with vN1, vΔN1 or vN1-rev at 2 p.f.u. per cell for 2 h followed by treatment with (black bars) or without (white bars) 1 μM ST for 2 h (panel a) or the indicated times (panel b), and were assayed for caspase activity (a), and caspase-3 (p17 fragment) and PARP (p89 fragment) cleavage (b). (c) HeLa cells were mock-infected or infected with vN1 or vΔN1 at 10 p.f.u. per cell for 6 h and mitochondrial (M), organelle (O) and cytosolic (C) fractions were isolated by subcellular fractionation. Fractions were analysed by immunoblotting for N1, cytochrome *c*, *α*-tubulin and Bcl-x_L_. Data are means±sd (**/^##^*P*<0.005, ***P<0.0005; Student's *t*-test, *n*=3). Blots are representative of three individual experiments.

To examine whether N1 displays anti-apoptotic activity we studied the cellular response to ST in cells transfected with a plasmid expressing N1 and in cells infected with viruses that do or do not express N1 (Methods). Transfection efficiencies as determined by MACS and FACS were between 30 and 50 %. Expression of either wild-type N1 or a mutant N1 protein with a C40S mutation (see below), by transfection, inhibited the ST-induced increase in mitochondrial membrane permeability (↑Δ*ψ*) and caspase 3/7 activation (Fig. 2a, b[Fig f2]). N1 was not as effective an inhibitor as Bcl-x_L_ possibly because N1 is a cytoplasmic protein (Bartlett *et al.*, 2002) whereas Bcl-x_L_ and Bcl-2 are targetted to mitochondria and the endoplasmic reticulum (ER) (discussed below). Therefore, N1 may prevent calcium release from the ER and mitochondrial apoptosis less effectively (Foyouzi-Youssefi *et al.*, 2000; Bassik *et al.*, 2004). Similarly, cells infected by a deletion mutant lacking N1 (vΔN1) (Bartlett *et al.*, 2002) had reduced ability to inhibit caspase 3/7 activity in response to ST (Fig. 3a[Fig f3]) compared with wild-type (vN1) and revertant (vN1-rev) controls. Also cleavage of caspase 3 and PARP were more rapid in vΔN1-infected cells (Fig. 3b[Fig f3]). However, compared with mock-infected cells, vΔN1-infected cells were still resistant to ST-induced apoptosis, consistent with the presence of other VACV apoptotic inhibitors (Taylor & Barry, 2006). Subcellular fractionation revealed that in vΔN1-infected cells the proportion of cytochrome *c* in the cytosol versus the mitochondria was increased compared with mock-infected and vN1-infected controls (Fig. 3c[Fig f3]). Analysis of *α*-tubulin levels verified equal loading.

### N1 binds to pro-apoptotic Bcl-2 family members

**Fig. 4. f4:**
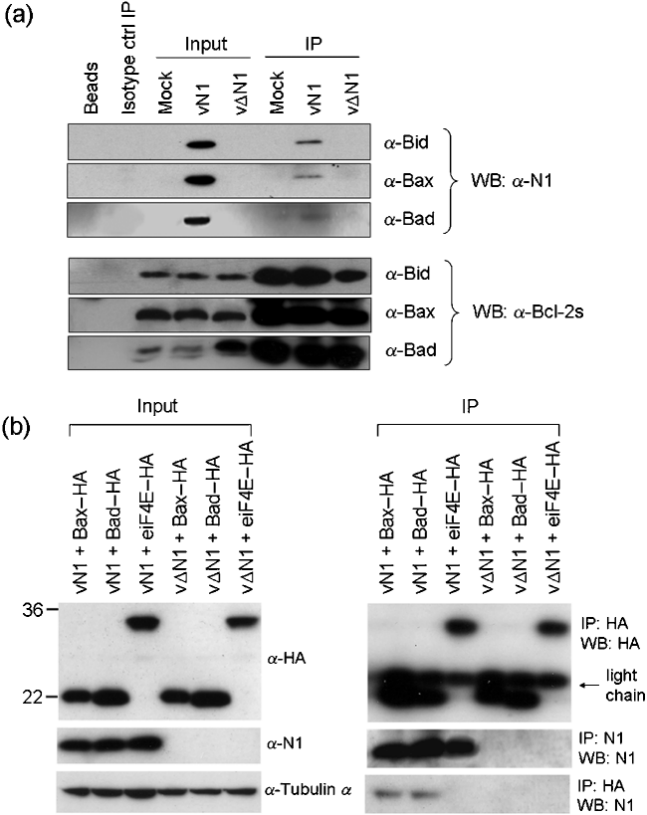
N1 interacts with Bad, Bax and Bid. Endogenous Bax, Bid and Bad (a) or overexpressed HA-tagged Bax, Bad or eiF4E control protein (b) were immunoprecipitated from lysates of HeLa cells infected with wild-type VACV (vN1) or N1 deletion virus (vΔN1). The input lysates and immunoprecipitates (IP) were fractionated by SDS-PAGE and immunoblotted for *α*-tubulin, N1, HA or Bax, Bid and Bad (Methods).

To address the mechanism by which N1 inhibits apoptosis, we investigated if N1 might interact with pro-apoptotic Bcl-2 family members. Cells were infected with vN1 or vΔN1 and extracts were immunoprecipitated with antibodies against Bad, Bax, Bid, Bak and Bim. Alternatively, cells were transfected with plasmids expressing HA-tagged Bad, Bax or control protein eiF4E, infected with vN1 or vΔN1 viruses, and immunoprecipitated with anti-HA or anti-N1 antibodies. Immunoprecipitates were analysed by immunoblotting with anti-N1 or anti-HA antibodies. N1 interacted with endogenous levels of Bad, Bax and Bid (Fig. 4a[Fig f4]), and also with overexpressed Bad and Bax (Fig. 4b[Fig f4]). An interaction between N1 and endogenous Bak or Bim was not detected (data not shown). A possible structural explanation for this selectivity is discussed below.

### Determination of N1 structure

**Fig. 5. f5:**
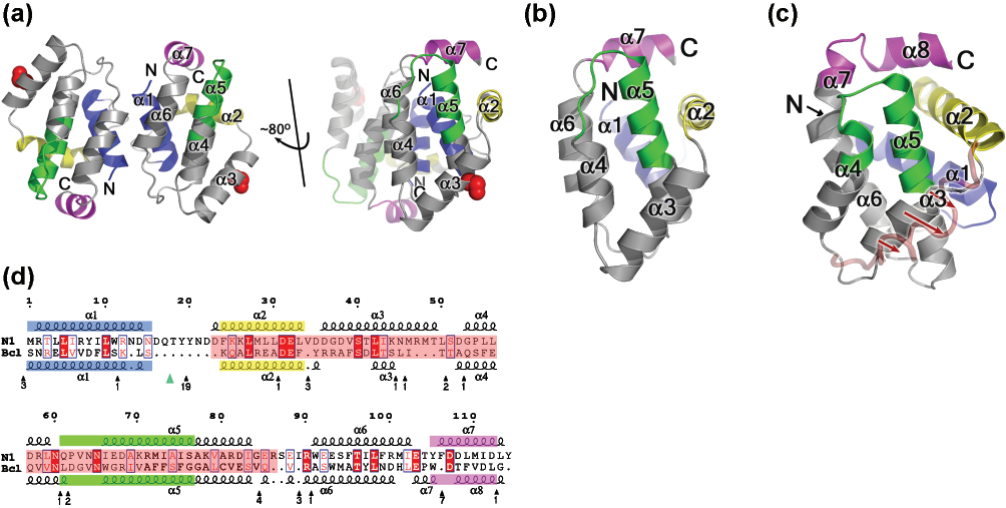
The structure of N1. (a) The dimer of N1 is viewed looking along the molecular twofold axis. BH1, BH2, BH3 and BH4 motifs are coloured green, magenta, yellow and blue, respectively and N and C termini are labelled. The right hand view is the same as in (b) and looks onto the surface groove and the mutated C40S residue is shown in red. For clarity, the helices of one monomer have been labelled. In the crystal structure of N1, the asymmetric unit has three dimers, each held together by anti-parallel interactions between helix *α*1 and *α*6 of each subunit, with a loss of 950 Å^2^ of solvent accessible surface. The interface does not involve the surface groove, which consequently is exposed and available to bind BH3 motifs. (b and c) Comparison of the structure of VACV N1 with Bcl-x_L_. Cartoons of N1 (b) and Bcl-x_L_ (c), with structurally equivalent BH1, BH2, BH3 and BH4 motifs coloured as in panel (a). The position adopted by helix 3 of Bcl-x_L_ when complexed with the Bim BH3 helix is drawn as a red semi-transparent loop and red arrows show movement of this helix upon peptide binding. (d) Sequence alignment of N1 and Bcl-x_L_ based on structural alignment from SHP (Stuart *et al.*, 1979). The core of the molecules, used in the structure based alignment, are highlighted in pink. Strictly conserved residues are in red blocks and similar residues in blue boxes. Secondary structural elements are coloured by BH motif definition, as above. To avoid breaking up the VACV N1 sequence, residues for Bcl-x_L_ that are not matched by N1 are omitted, and the position and number of residues removed are indicated under the Bcl-x_L_ sequence. The position of a disordered loop in Bcl-x_L_ is marked by the green triangle.

To investigate how N1 was able to inhibit apoptosis and bind pro-apoptotic Bcl-2 family members, its crystal structure was determined. The full-length wild-type N1 and a C40S mutant were expressed in *E. coli*, purified, crystallized and the structure was solved at 2.9 Å resolution by MAD analysis of the selenomethionated protein (Methods and Table 1). The C40S mutation was introduced to avoid intermolecular disulphide bond driven aggregation. The crystal contains three dimers per asymmetric unit. The structural model encompasses residues 1–114 (residues 115–7 were not visible in the electron density map) and has an R_factor_/R_free_ of 23.7/24.4 %. The structure of the dimer is shown in Fig. 5(a). Note that the C40S substitution is distal to the dimer interface.

### The overall topology of N1 is similar to Bcl-x_L_

N1 contains seven *α*-helices (Fig. 5b[Fig f5]) arranged with a fold similar to Bcl-x_L_ (Fig. 5c[Fig f5]) (Muchmore *et al.*, 1996) and other Bcl-2 family members (Chou *et al.*, 1999; Suzuki *et al.*, 2000; Petros *et al.*, 2001; Hinds *et al.*, 2003; Day *et al.*, 2005), including viral counterparts (Huang *et al.*, 2002, 2003; Polster *et al.*, 2004; Loh *et al.*, 2005). Helix *α*5 forms the hydrophobic core of the structure and is surrounded by the other helices. Comparison of N1 with Bcl-x_L_ using program SHP (Stuart *et al.*, 1979) matched 96 C*α*s out of 114 with root-mean-square deviation (rmsd) 2.4 Å, and revealed secondary structure elements representing the BH motifs of Bcl-2 family members equivalent to those in Bcl-x_L_ (Fig. 5d[Fig f5]). These similarities were not apparent from sequence alignments. Although the overall fold of Bcl-2 family members is conserved, there are notable differences mainly in the relative lengths of the helices and connecting loops. The *α*1–*α*2 loop of N1 is considerably shorter (8 residues) than in Bcl-x_L_ (63 residues), a feature shared with other viral Bcl-2 counterparts (Huang *et al.*, 2003; Polster *et al.*, 2004; Loh *et al.*, 2005). In cellular Bcl-2 family members, this loop contains sites for caspase cleavage and phosphorylation (Polster *et al.*, 2004), which converts anti-apoptotic Bcl-2 proteins into pro-apoptotic molecules. This structural alteration is an example of how viral proteins have evolved to circumvent cellular regulatory mechanisms. Other differences include the positions of helices *α*3 and *α*4, which lie roughly anti-parallel in the ligand-free structure of Bcl-x_L_, but are almost orthogonal in N1. This change affects the surface topology of N1, producing a groove, which is discussed below. Bcl-x_L_ contains a longer *α*5 helix and shorter *α*5–*α*6 loop than N1. Furthermore, *α*6 runs into an extra helix in Bcl-x_L_, whilst in N1 the structure is shortened, a compact loop enabling interaction between *α*6 and *α*7. The C-terminal hydrophobic *α*-helix of Bcl-x_L_, Bcl-w and Bax localizes these Bcl-2 proteins to mitochondria where they regulate the permeability of the outer mitochondrial membrane (Martinou & Green, 2001), and prevention of calcium release from the ER (Foyouzi-Youssefi *et al.*, 2000; Bassik *et al.*, 2004). This helix is lacking in N1 and, consistent with this, N1 is cytosolic (Bartlett *et al.*, 2002) and does not change location after pro-apoptotic stimulation or co-localize with mitochondria in apoptotic cells (data not shown).

### Properties of the N1 BH3-binding groove and dimer interface

**Fig. 6. f6:**
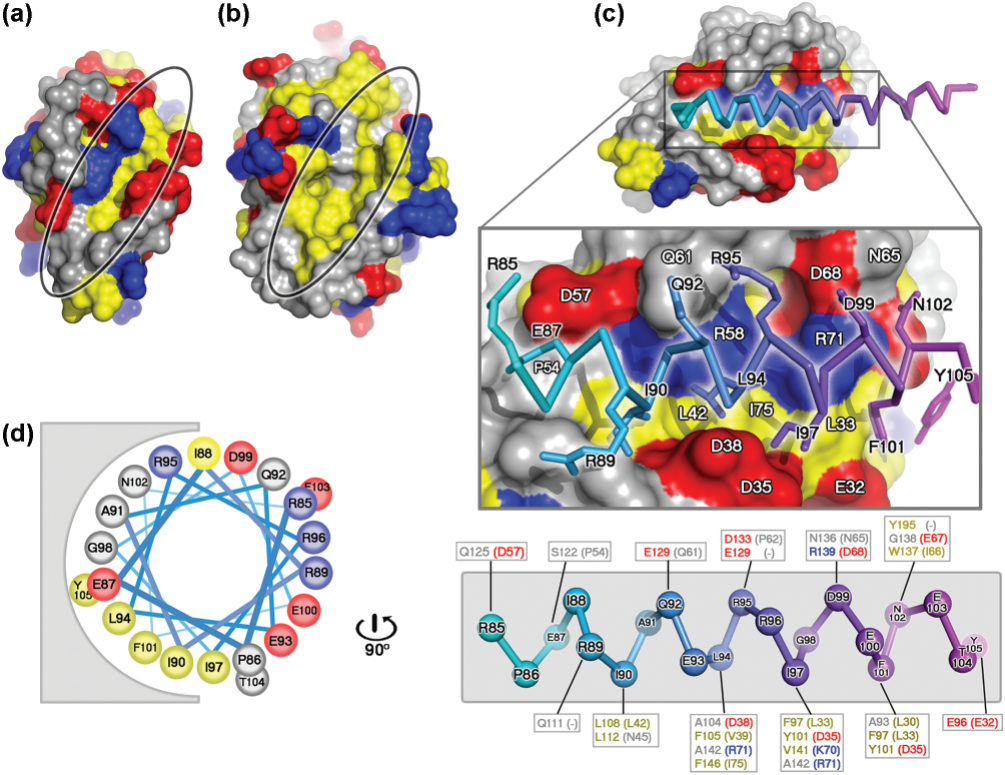
Comparison of N1 and Bcl-x_L_ surface grooves and molecular modelling of BH3 peptides. Molecular surfaces of N1 (a) and Bcl-x_L_ (b), with the grooves highlighted. Lys, Arg and His are coloured in blue, Asp and Glu are coloured in red and Leu, Ile, Val, Met, Tyr, Phe and Trp are coloured in yellow. All other residue types are coloured in grey. (c) Molecular surface of N1 with the Bim BH3 helix (PDB code 1PQ1) modelled into the surface groove as described in the text. The surface of N1 is coloured as described in (a). The peptide is coloured from cyan to magenta from the N to the C terminus. The middle panel shows the Bim peptide from residue 85 to 105 with contacting residues drawn in atomic detail. Residues of the peptide contacting N1 are labelled in black and residues of N1 contacting the peptide are labelled in white. The lower panel shows the Bim BH3 peptide helix skeleton coloured from cyan to magenta, with the contacting residues of Bcl-x_L_ and their equivalents of N1 boxed (N1 residues are in parentheses), and residues coloured according to their properties, as in (a). (d) Helical wheel representation of the Bim BH3 peptide (residues 85–105), drawn rotating the peptide in (c) by 90 ° around an axis vertical to the page, showing the predominantly amphipathic nature of the BH3 helix.

The predominantly acidic surface of N1 (Fig. 6a[Fig f6]) contains a surface groove formed by helices *α*2, *α*3, *α*4 and the N terminus of *α*5 (Fig. 5b[Fig f5]). In anti-apoptotic Bcl-2 family members, corresponding hydrophobic grooves serve as binding sites for the BH3 motifs of pro-apoptotic members (Sattler *et al.*, 1997; Petros *et al.*, 2000; Liu *et al.*, 2003). For Bcl-x_L_, unbound and peptide bound structures (Liu *et al.*, 2003) reveal that on peptide binding helix *α*3 is displaced, opening the groove to accommodate the peptide (Fig. 5c[Fig f5]). In contrast, the N1 groove has an open conformation that could readily bind BH3 regions of pro-apoptotic Bcl-2 proteins. Notably, N1 superimposes better on the Bcl-x_L_–Bim open-groove conformation (rmsd 2.4 Å) than the unbound closed-groove conformation (rmsd 2.8 Å). In Bcl-x_L_, residues from the BH1, BH2 and BH3 motifs are crucial for binding peptides from Bad (Petros *et al.*, 2000), Bak (Sattler *et al.*, 1997) and Bim (Liu *et al.*, 2003), usually by forming hydrophobic pockets in the groove. In N1, two of these hydrophobic residues (L27 and L42, located in *α*2 and *α*3) are conserved (Bcl-x_L_ residues L90 and L108, respectively), some others are similar (L30–A93, L33–F97, V39–F105, I66–W137 and I75–F146; the first residue in each pair is that of N1), whereas others are charged (D38–A104, R58–V126, K70–V141 and R71–A142). Overall, the N1 groove is distinguished by these charged residues (Fig. 6a–c[Fig f6]) and by the absence of the characteristic NWGR sequence found at the N terminus of *α*5 in other Bcl-2 structures (Petros *et al.*, 2004) [whilst the N is conserved (N65) and W is replaced by the chemically similar I66, the GR pair are replaced by acidic residues E67 and D68, Fig. 5d[Fig f5]]. These changes suggest that contacts between the N1 groove and BH3 ligands would differ from the paradigm provided by the Bcl-x_L_–Bim peptide complex (Liu *et al.*, 2003). Residues I, L, I and F at points 1, 5, 8 and 12 in the BH3 motif (Figs 6d[Fig f6], 7) provide key anchor points in the hydrophobic Bcl-x_L_ pockets (Figs 6b[Fig f6], 7). These anchors appear generic; similar hydrophobic residues being used in binding to Bak and Bad peptides (Sattler *et al.*, 1997; Petros *et al.*, 2000) (position 5 is strictly conserved).

The dimers of N1 observed in the crystal structure are held together by anti-parallel interactions between *α*1 and *α*6 (Fig. 5a[Fig f5]). The constitutively open hydrophobic surface grooves are distal to this dimer interface, allowing the dimer to readily bind BH3 motifs of pro-apoptotic proteins. The N1 dimer interface differs markedly from the only other dimeric Bcl-2 protein reported, for Bcl-x_L_, which shows a domain-swapped dimeric association via the loop connecting *α*5 and *α*6 (O'Neill *et al.*, 2006). In the N1 dimer, no such domain exchange exists. The ability of N1 to function as a homodimer (Bartlett *et al.*, 2002) suggests that it might bind to two pro-apoptotic Bcl-2s simultaneously.

### Charged residues in the N1-binding groove may contribute to BH3-binding specificity

**Fig. 7. f7:**
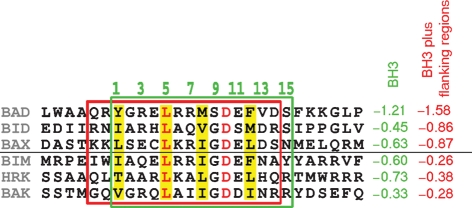
The hydrophobic properties of the BH3 motifs (boxed in green) and flanking regions (boxed in red) of pro-apoptotic Bcl-2 proteins Bad, Bid, Bax, Bim, Hrk and Bak are shown as GRAVY (GRand AVerage of hydropathY) scores (Kyte & Doolittle, 1982). The numbers above the alignment denote the residues in the BH3 motif, and the conserved hydrophobic residues are coloured yellow.

To investigate if N1 might bind BH3 peptides, we first modelled Bad, Bak and Bim peptides into the N1 groove. Structural superposition (Stuart *et al.*, 1979) of N1 and the mouse Bcl-x_L_–Bim complex positioned the Bim peptide into the N1 groove. The Bad and Bak peptides were then superposed on Bim. This modelling procedure resulted in minor steric clashes between the peptides and the groove (e.g. R71 with G98 of the Bim peptide) that could be relieved by slight rearrangements. In each model, the central portion of the groove is occupied by the core sequence motif of the peptides representing the conserved BH3 motif (Fig. 6c[Fig f6]). However, the pockets that locate the hydrophobic anchor residues (which are entirely hydrophobic in Bcl-x_L_) are, in N1, lined by both hydrophobic and charged residues (for instance D38, V39, R71 and I75 for the L94 pocket, Fig. 6c[Fig f6]). As the N1 groove is constitutively open, such changes may be a strategy to avoid the unfavourable thermodynamic consequences of exposed hydrophobic patches on the protein surface. Collectively, these observations predict that N1 might bind the pro-apoptotic Bcl-2 proteins via their BH3 motifs, consistent with the biochemical data presented above (Fig. 4). Furthermore, the BH3 motifs of the pro-apoptotic Bcl-2 proteins Bad, Bax and Bid are markedly less hydrophobic than those of Bak, Bim and Hrk due to differences in the residues flanking the core BH3 motif (Fig. 7). This difference might explain why interactions between N1 and endogenous Bad, Bax and Bid, but not Bak or Bim were detected by immunoprecipitation. In contrast, *in vitro* binding of N1 to BH3 peptides from Bid, Bim and Bak, but not Bad, was reported by Aoyagi *et al.* (2007), but these interactions were not confirmed by analysis of whole proteins. These differences may reflect the different methods used. It is possible that, like N1, cellular Bcl-2 proteins also achieve selectivity by sensing the sequence of BH3 motif flanking regions.

### Conclusions

Functional analysis demonstrates that N1 inhibits apoptosis both in transfected cells and in VACV-infected cells and, consistent with this, biochemical analysis shows that N1 binds pro-apoptotic Bcl-2 family proteins Bad, Bax and Bid. The crystal structure of N1 reveals that it is a dimeric Bcl-2-like protein with a surface groove that is constitutively open and that is predicted by molecular modelling to bind BH3 motif peptides of pro-apoptotic Bcl-2 family members. The surface groove also indicates how N1 might bind BH3 peptides selectively, and this has implications for cellular Bcl-2 protein interactions. The lack of significant sequence similarity between N1 and other Bcl-2 family proteins suggests that retention of the structure is a result of strong evolutionary pressure, and underlines the importance of structural information in understanding the molecular functions of proteins. It remains possible that other viral or cellular Bcl-2 family proteins exist that will only be identified through structural analysis. N1 is the first VACV protein shown to be a Bcl-2 family member and represents another example of VACV anti-apoptotic proteins.

## Figures and Tables

**Table 1. t1:**
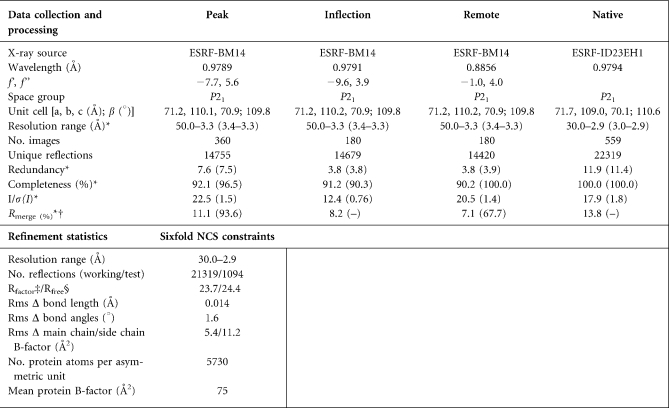
Summary of crystallographic statistics; data collection, processing and refinement

*Values in parentheses are for the outermost resolution range. †*R*_merge_=Σ_hkl_Σ_i_|*I*_hkl;i_–<*I*_hkl_>|/Σ_hkl_Σ_i_*I*_hkl;i_, where *I*_hkl;i_ is the intensity of an individual measurement and <*I*_hkl_>is the mean intensity from multiple observations. ‡R_factor_=Σ_hkl_||*F*obs|_hkl_−*k*|*F*calc|_hkl_|/Σ_hkl_|*F*obs|_hkl_. §R_free_ equals the R_factor_ against 5 % of the data removed prior to refinement. Rms Δ, root-mean-square deviation from ideal geometry.
